# Altered functional connectivity of the cingulate subregions in schizophrenia

**DOI:** 10.1038/tp.2015.69

**Published:** 2015-06-02

**Authors:** D Wang, Y Zhou, C Zhuo, W Qin, J Zhu, H Liu, L Xu, C Yu

**Affiliations:** 1 Department of Radiology and Tianjin Key Laboratory of Functional Imaging, Tianjin Medical University General Hospital, Tianjin, China; 2 Tianjin Anding Hospital (Tianjin Mental Health Center), Tianjin, China; 3 Tianjin Anning Hospital, Tianjin, China

## Abstract

Schizophrenia patients have shown altered resting-state functional connectivity (rsFC) of the cingulate cortex; however, it is unknown whether rsFCs of the cingulate subregions are differentially affected in this disorder. We aimed to clarify the issue by comparing rsFCs of each cingulate subregion between healthy controls and schizophrenia patients. A total of 102 healthy controls and 94 schizophrenia patients underwent resting-state functional magnetic resonance imaging with a sensitivity-encoded spiral-in imaging sequence to reduce susceptibility-induced signal loss and distortion. The cingulate cortex was divided into nine subregions, including the subgenual anterior cingulate cortex (ACC), areas 24 and 32 of the pregenual ACC, areas 24 and 32 of the anterior mid-cingulate cortex (aMCC), posterior MCC (pMCC), dorsal (dPCC) and ventral (vPCC) posterior cingulate cortex (PCC) and retrosplenial cortex (RSC). The rsFCs of each cingulate subregion were compared between the two groups and the atrophy effect was considered. Results with and without global signal regression were reported. Most cingulate subregions exhibited decreased rsFCs in schizophrenia after global signal regression (GSR). Without GSR, only increased rsFC was found in schizophrenia, which primarily restricted to the aMCC, PCC and RSC. Some of these increased rsFCs were also significant after GSR. These findings suggest that GSR can greatly affect between-group differences in rsFCs and the consistently increased rsFCs may challenge the functional disconnection hypothesis of schizophrenia.

## Introduction

Schizophrenia is a severe psychiatric condition characterized by hallucination, delusion and impaired perception and cognition,^1, 2^ which have been attributed to structural and functional alterations of the brain, including the cingulate cortex.^3, 4, 5^ Schizophrenia patients have exhibited abnormalities in cortical thickness,^6^ gray matter volume (GMV),^7^ metabolism,^8^ cerebral blood flow,^9^ task-evoked activation,^10^ spontaneous activity,^11^ anatomical connection^12^ and resting-state functional connectivity (rsFC) ^13^ in the cingulate cortex. However, the cingulate cortex is a structurally^14^ and functionally^15^ heterogeneous region. On the basis of integrated neurobiological assessments, it has been roughly subdivided into seven subregions, including the subgenual (sACC) and pregenual (pACC) parts of the anterior cingulate cortex (ACC), the anterior (aMCC) and posterior (pMCC) parts of the mid-cingulate cortex (MCC), the dorsal (dPCC) and ventral (vPCC) parts of the posterior cingulate cortex (PCC), and the retrosplenial cortex (RSC).^16^ According to cytoarchitectonic features, the pACC and aMCC can be further subdivided into two subregions, that is, the area 24 and the area 32. Thus a total of nine subregions could be identified for each side of the cingulate cortex.

Extensive rsFC reduction has been reported in schizophrenia patients, highlighting the role of functional disconnection in the pathophysiology of the disorder.^17, 18, 19, 20^ As for the cingulate cortex, most previous rsFC studies have focused on connectivity changes of the PCC and ACC; however, rsFC changes of other cingulate subregions in schizophrenia remain largely unknown. Even for the PCC and ACC, inconsistent findings (either increase or decrease) have been reported about rsFC changes of these two subregions in schizophrenia. Moreover, these studies investigated rsFC changes of the PCC and ACC in schizophrenia from the perspectives of specific functional networks, such as the default mode network (DMN) for the PCC^21, 22^ and the salience network (SN) for the ACC.^23^ There is lack of a systematical investigation on the rsFC changes of the cingulate subregions in schizophrenia.

Most of the previous studies on rsFC have used echo-planar imaging (EPI) technique, which is challenged by susceptibility-induced signal loss and distortion in the sACC and orbitofrontal cortex. To reduce susceptibility artifacts in air/tissue interfaces, a sensitivity-encoded spiral imaging (SENSE-SPIRAL) technique has been proposed to acquire functional magnetic resonance imaging (fMRI) data.^24^ This technique may improve the image quality of the sACC and make rsFC analysis of this region more reliable than the EPI sequence.

In this study, we aimed to systematically investigate the rsFC differences of each cingulate subregion between schizophrenia patients and healthy controls using the fMRI data acquired by the SENSE-SPIRAL sequence. We hypothesize that cingulate subregions are affected differentially in schizophrenia. Because recent studies report that global signal regression affects results of rsFC comparisons between schizophrenia patients and healthy controls,^25, 26, 27^ we analyzed the fMRI data with and without global signal regression.

## Materials and methods

### Participants

This study included 94 schizophrenia patients and 102 healthy controls. Diagnoses for patients were confirmed using the Structured Clinical Interview for DSM-IV. Inclusion criteria were age (16–60 years) and right-handedness. Exclusion criteria were MRI contraindications, poor image quality, presence of a systemic medical illness or central nervous system disorder, history of head trauma, substance abuse within the last 3 months or lifetime history of substance abuse or dependence. Additional exclusion criteria for healthy controls were history of any Axis I or II disorders and a psychotic disorder and first-degree relative with a psychotic disorder. The Positive and Negative Syndrome Scale was used to quantify the clinical symptoms.^28^ The positive subscores measure delusion, conceptual disorganization, hallucinatory behavior, excitement, grandiosity, suspiciousness and hostility; and the negative subscores measure blunted affect, emotional withdrawal, poor rapport passive-apathetic social withdrawal, difficulty in abstract thinking, lack of spontaneity and flow of conversation, and stereotyped thinking.^28^ This study was approved by the Medical Research Ethics Committee of Tianjin Medical University General Hospital, and all the participants provided written informed consent.

### Imaging data acquisition

MRI data were acquired by a 3.0 Tesla MR system (Discovery MR750, General Electric, Milwaukee, WI, USA). Tight but comfortable foam padding was used to minimize head motion, and earplugs were used to reduce scanner noise. Sagittal three-dimensional T1-weighted images were acquired by a brain volume sequence with the following scan parameters: repetition time (TR)=8.2 ms; echo time (TE)=3.2 ms; inversion time (TI)=450 ms; flip angle (FA)=12° field of view (FOV)=256 mm × 256 mm; matrix=256 × 256; slice thickness=1 mm, no gap; and 188 sagittal slices. Two sets of resting-state fMRI data were acquired. A gradient-echo single-shot EPI sequence was performed using parameters of TR/TE=2000/45 ms; FOV=220 mm × 220 mm; matrix=64 × 64; FA=90° slice thickness=4 mm; gap=0.5 mm; 32 interleaved transverse slices; 180 volumes. A gradient-echo SENSE-SPIRAL (spiral-in) sequence was performed using parameters of TR/TE=1400/30 ms; FA=60°, acceleration factor=2, and dummy scan for the first 10 time points. The FOV, matrix, slice thickness, gap and slice number were the same as the EPI sequence. During these fMRI scans, all the subjects were instructed to keep their eyes closed, to relax and move as little as possible, to think of nothing in particular and to not fall asleep.

### GMV calculation

The GMV of each voxel was calculated using Statistical Parametric Mapping software (SPM8; http://www.fil.ion.ucl.ac.uk/spm/software/spm8/). The structural MR images were segmented into gray matter (GM), white matter and cerebrospinal fluid using the standard unified segmentation model. After an initial affine registration of GM concentration map into the Montreal Neurological Institute (MNI) space, GM concentration images were nonlinearly warped using diffeomorphic anatomical registration through the exponentiated Lie algebra technique^29^ and were resampled to 1.5-mm cubic voxels. The GMV of each voxel was obtained by multiplying GM concentration map by the nonlinear determinants derived from the spatial normalization step. Finally, GMV images were smoothed with a Gaussian kernel of 6 × 6 × 6 mm^3^ full-width at half maximum. After spatial preprocessing, the normalized, modulated, and smoothed GMV maps were used for statistical analysis.

### fMRI data preprocessing

Two sets of resting-state fMRI data were preprocessed using the SPM8 with the same procedures. The first 10 volumes for each subject were discarded to allow the signal to reach equilibrium and the participants to adapt to the scanning noise. The remaining volumes were then corrected for the acquisition time delay between slices. All subjects' fMRI data were within defined motion thresholds (translational or rotational motion parameters less than 2 mm or 2°). We also calculated framewise displacement, which indexes volume-to-volume changes in head position.^30^ Considering recent studies reported that signal spike caused by head motion significantly contaminated the final resting-state fMRI results even after regressing out the realignment parameters,^30^ we removed spike volumes when the framewise displacement of specific volume exceeded 0.5. After regressing out the effects of six motion parameters, we used a component-based noise correction method (COMPCOR) to regress out the average BOLD signals of the ventricular and white matter, and the physiological noise.^31^ During this step, we remained both the preprocessed fMRI data with and without GSR. The datasets were band-pass filtered with a frequency range of 0.01 to 0.08 Hz. Individual structural images were linearly coregistered to the mean functional image; then the transformed structural images were segmented into GM, white matter, and cerebrospinal fluid. The GM maps were linearly coregistered to the tissue probability maps in the MNI space. Finally the motion-corrected functional volumes were spatially normalized to the MNI space using the parameters estimated during linear coregistration. The functional images were resampled into 3 × 3 × 3 mm^3^ voxels. After normalization, all data sets were smoothed with a Gaussian kernel of 6 × 6 × 6 mm^3^ full-width at half maximum.

### Functional connectivity analysis

The regions of interest (ROIs) for the rsFC analyses were defined according to a previous study;^16^ in that study, the human cingulate cortex was divided into nine ROIs, that is, the ROI 1 (sACC), ROI 2 (area 24 of the pACC), ROI 3 (area 32 of the pACC), ROI 4 (area 24 of the aMCC), ROI 5 (area 32 of the aMCC), ROI 6 (pMCC), ROI 7 (dPCC), ROI 8 (vPCC) and ROI 9 (RSC). After these ROIs were normalized into the MNI template, we conducted ROI-based rsFC analyses with the following procedures.

For each individual data set, Pearson's correlation coefficients between the mean time series of each ROI and time series of each voxel in other parts of the brain were computed and converted to *z*-values using Fisher's *r-*to-*z* transformation to improve the normality. Then, individuals' *z*-values were entered into a random-effect one-sample *t*-test in a voxel-wise manner using the SPM8. A false discovery rate correction with *P*<0.05 was used to identify brain regions that showed significant positive correlations with each ROI. Then a two-sample *t*-test was performed within the positive rsFC mask to quantitatively compare differences in rsFC of each ROI between healthy controls and schizophrenia patients when controlling for age and gender. A false discovery rate correction with *P*<0.05 was used to identify the statistical significance of these comparisons.

### GMV analysis

We extracted GMV of each cingulate subregion and compared between the two groups using a two-sample *t*-test. Multiple comparisons were corrected using the Bonferroni method with a significant threshold of *P*<0.05/18=0.003.

### Effects of GMV atrophy of the cingulate subregions on their rsFC changes

To test the effect of GMV atrophy on the rsFC changes of the cingulate subregions, we extracted each rsFC of the cingulate subregions with significant difference between the two groups in the voxel-based analysis. Then we used general linear model to compare intergroup differences in these rsFCs without and with GMV correction. GMV correction was realized by adding GMV of each cingulate subregion as a covariant of no interest.

### Correlations between rsFCs of the cingulate subregions and clinical parameters

To test whether rsFCs of the cingulate subregions with significant group differences were correlated with clinical variables, we extracted these rsFCs and calculated Spearman's correlation coefficients between these rsFCs and clinical parameters (that is, Positive and Negative Syndrome Scale, illness duration and antipsychotic dosage; *P*<0.05, uncorrected).

## Results

### Demographic and clinical characteristics

Demographic and clinical characteristics of schizophrenia patients and healthy controls are summarized in Table 1. With the exception of six individuals who were not receiving antipsychotic medication, patients were receiving treatment with one (*n*=63) or more (*n*=26) atypical antipsychotics, and one patient cannot remember the antipsychotics clearly. The two groups did not differ in gender (*χ*^2^=2.46, df=1, *P*=0.12) and age (*t*=0.15, df=194, *P*=0.88).

### Assessments of signal intensity and distortion of the sACC

**Figure 1:**
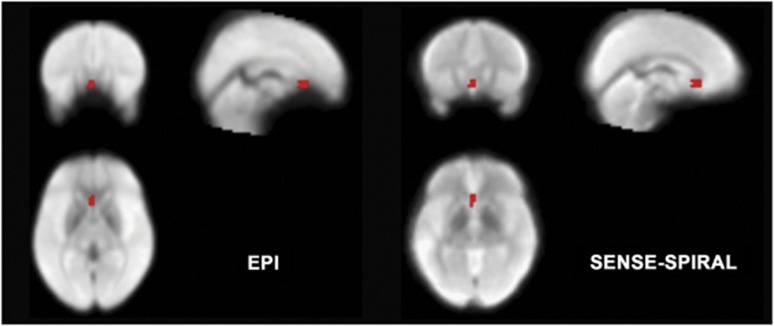
Distortion and signal loss of functional images derived from various acquisition sequences. The temporal pole, subgenual anterior cingulate cortex (red color) and orbitofrontal cortex exhibit less distortion and signal loss in fMRI data acquired by the sensitivity-encoded spiral-in imaging (SENSE-SPIRAL) sequence (**a**) than in those acquired by the echo-planar imaging (EPI) sequence (**b**). fMRI, functional magnetic resonance imaging.

We projected the ROI of the sACC onto the mean normalized template of the fMRI data derived from the EPI and SENSE-SPIRAL sequences to compare signal loss and distortion of the sACC between the two acquisition sequences. Compared with those derived from the EPI sequence, the sACC images derived from the SENSE-SPIRAL sequence exhibited less signal loss and distortion (Figure 1). Therefore, we chose fMRI data acquired by the SENSE-SPIRAL sequence for rsFC analyses.

### The rsFC patterns of the cingulate subregions

**Figure 2:**
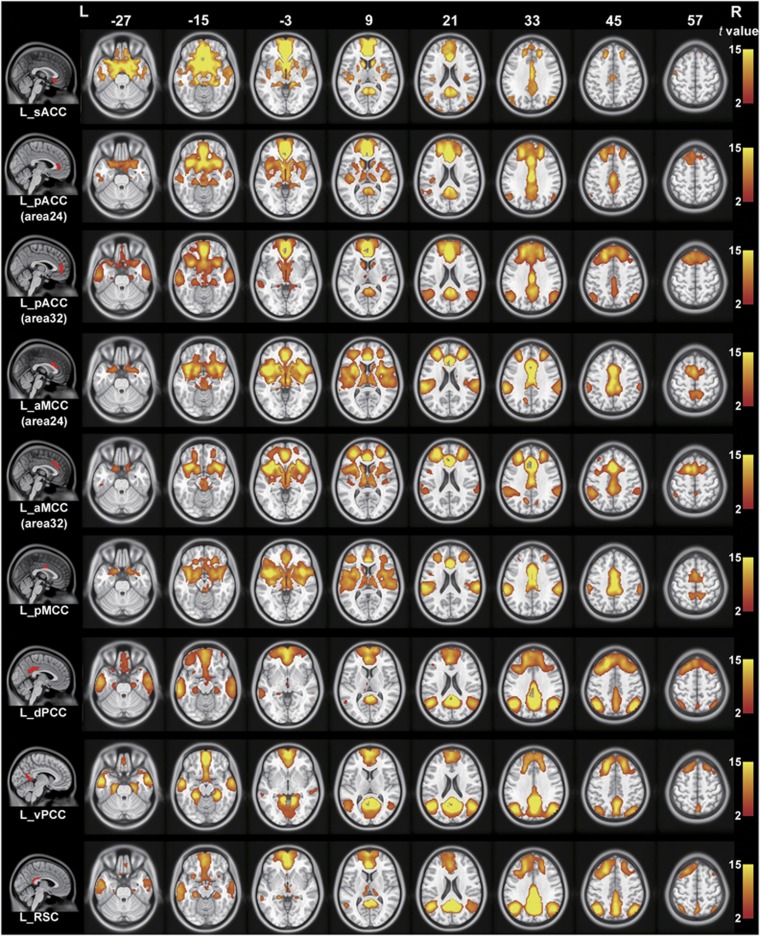
Resting-state functional connectivity maps of the left cingulate subregion in healthy controls with global signal regression. Only positive connectivity map of each cingulate subregion is depicted. Multiple comparisons are corrected by a false discovery rate (FDR) with a significant threshold of *P*<0.05. aMCC, anterior mid-cingulate cortex; dPCC, dorsal posterior cingulate cortex; L, left; pACC, pregenual anterior cingulate cortex; pMCC, posterior mid-cingulate cortex; R, right; RSC, retrosplenial cortex; sACC, subgenual anterior cingulate cortex; vPCC, ventral posterior cingulate cortex.

**Figure 3:**
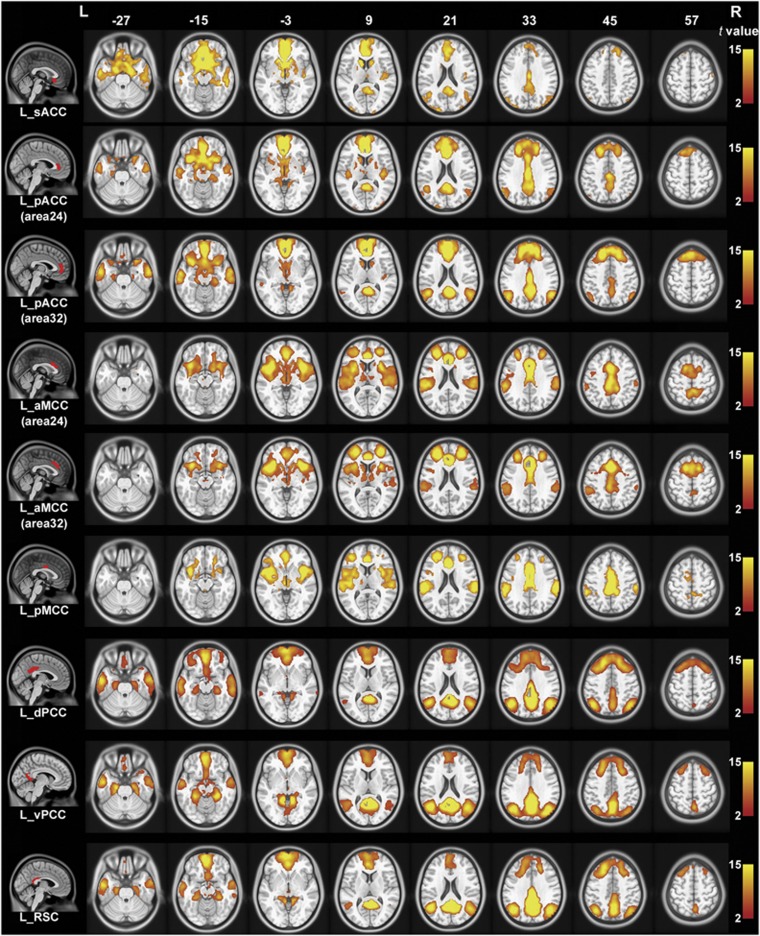
Resting-state functional connectivity maps of the left cingulate subregion in schizophrenia controls with global signal regression. Only positive connectivity map of each cingulate subregion is depicted. Multiple comparisons are corrected by a false discovery rate (FDR) with a significant threshold of *P*<0.05. aMCC, anterior mid-cingulate cortex; dPCC, dorsal posterior cingulate cortex; L, left; pACC, pregenual anterior cingulate cortex; pMCC, posterior mid-cingulate cortex; R, right; RSC, retrosplenial cortex; sACC, subgenual anterior cingulate cortex; vPCC, ventral posterior cingulate cortex.

With GSR, the rsFC maps of the left cingulate subregions are shown in Figure 2 for healthy controls and in Figure 3 for schizophrenia patients; the rsFC maps of the right cingulate subregions are shown in Supplementary Figures 1 and 2. Both healthy controls and schizophrenia patients exhibited similar rsFC patterns for each cingulate subregion; however, the patient group had a smaller spatial extent in brain regions that exhibited significant rsFCs with the sACC, pACC, aMCC and pMCC.

The sACC (ROI 1) was mainly correlated with brain regions of the affective network (AN), including the medial prefrontal cortex (MPFC), orbitofrontal cortex (OFC) and temporal pole. The pACC (ROI 2 and ROI 3) was correlated with the AN, the DMN including the PCC, MPFC, lateral parietal cortex and anterior temporal cortex, and the SN including the aMCC, fronto-insular cortex and striatum. The aMCC (ROI 4 and ROI 5) was correlated with the SN and the sensorimotor network including the sensorimotor cortex, supplementary motor area (SMA), thalamus and striatum. The pMCC (ROI 6) showed similar connectivity patterns to the aMCC; however, it had stronger connectivity with the sensorimotor network and weaker connectivity with the SN. The PCC (ROI 7 and ROI 8) and RSC (ROI 9) were mainly correlated with the DMN.

### Group differences in rsFCs of the cingulate subregions

Group differences in rsFCs of the cingulate subregions with GSR are shown in Supplementary Table 1 and Supplementary Figures 3 and 4. Compared with healthy controls, schizophrenia patients showed significantly reduced rsFCs of the bilateral sACC, pACC (area 24), aMCC (area 24 and 32), pMCC and RSC, and the left pACC (area 32). The increased rsFC were observed in the right aMCC (area 24) and dPCC, and bilateral vPCC and RSC.

In schizophrenia, the bilateral sACC had decreased connectivity with the ventral MPFC. The left sACC had decreased connectivity with the right temporal pole. The right sACC also had decreased connectivity with the bilateral amygdala, aMCC, pMCC and right insular cortex. The bilateral pACC (area 24) showed decreased connectivity with the bilateral fronto-insular cortex, aMCC and left striatum. The right pACC (area 24) also showed decreased connectivity with the left OFC. The left pACC (area 32) had decreased connectivity with the left aMCC and PFC. The bilateral aMCC (area 24) showed decreased connectivity with the striatum, thalamus, insular cortex, pACC and pMCC. The right aMCC (area 24) also showed decreased connectivity with bilateral SMA and increased connectivity with the left postcentral gyrus. The left aMCC (area 32) had reduced connectivity with the bilateral striatum, thalamus, insular cortex, pACC and dPCC, left middle frontal gyrus and precuneus, and right aMCC. The right aMCC (area 32) showed decreased connectivity with the bilateral angular gyrus, middle frontal gyrus, thalamus and MPFC, left striatum, right OFC and right pMCC. The left pMCC showed decreased connectivity with the bilateral SMA, insular cortex, striatum, pACC, aMCC and temporal pole, and right OFC, thalamus and amygdala. The right pMCC had reduced connectivity with the bilateral SMA, thalamus, striatum, insular cortex, pACC, aMCC, and left pMCC. The right dPCC showed increased connectivity with right lingual gyrus. The left vPCC had increased connectivity with the right middle occipital gyrus. The right vPCC showed increased connectivity with the bilateral precuneus. The left RSC had increased connectivity with the right lingual gyrus and reduced connectivity with the left superior parietal gyrus. The right RSC showed decreased connectivity with the left MPFC and increased connectivity with bilateral lingual gyrus.

Group differences in rsFCs of the cingulate subregions without GSR are shown in the Supplementary Figure 5 and Supplementary Table 2. Compared with healthy controls, schizophrenia patients showed increased rsFCs of the bilateral aMCC (area 24), dPCC, vPCC and RSC. The bilateral aMCC had increased connectivity with the left postcentral gyrus. The left dPCC had increased connectivity with the bilateral lingual gyrus. The right dPCC had increased connectivity with the right lingual gyrus. The left vPCC had increased connectivity with the right precuneus, middle occipital gyrus, MCC and middle frontal gyrus. The right vPCC had increased connectivity with the bilateral precuneus and middle occipital gyrus. The left RSC had increased connectivity with the bilateral parahippocampal gyrus, left OFC and lingual gyrus, and right middle temporal gyrus, superior frontal gyrus, calcarine gyrus, precuneus and middle frontal gyrus. The right RSC had increased connectivity with the bilateral parahippocampal gyrus, left superior frontal gyrus, right lingual gyrus and middle frontal gyrus.

**Figure 4:**
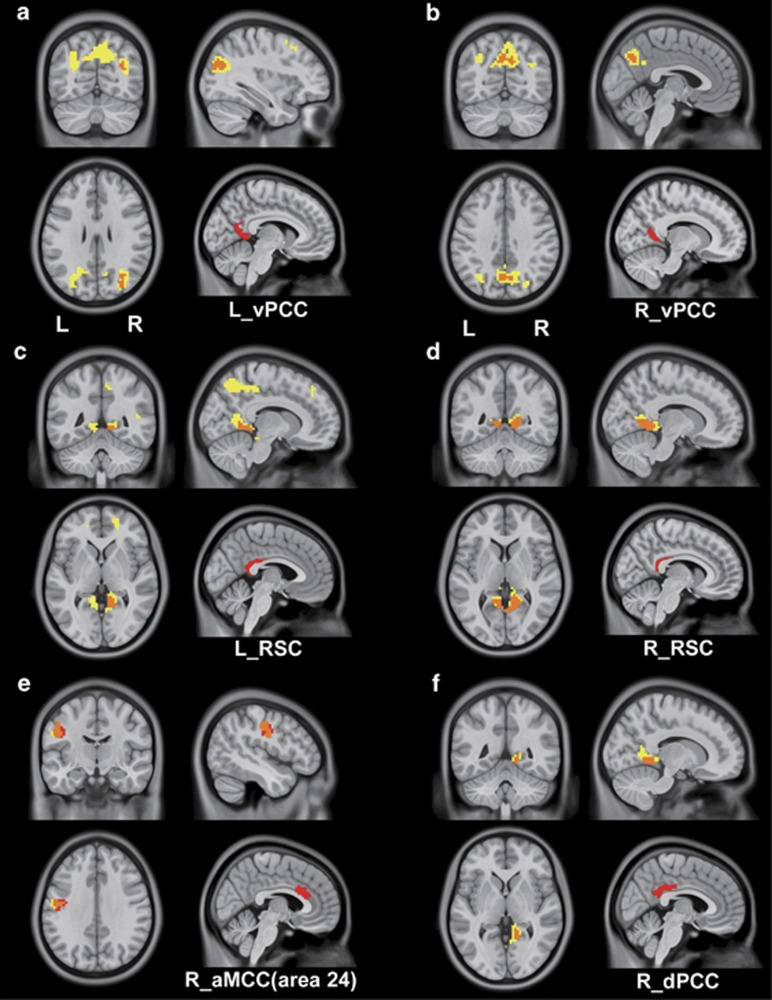
The consistent results of between-group differences with and without global signal regression (GSR). Red color represents increased connectivity with GSR and yellow color indicates increased connectivity without GSR in schizophrenia patients, the orange color represents the overlapping. aMCC, anterior mid-cingulate cortex; dPCC, dorsal posterior cingulate cortex; L, left; R, right; RSC, retrosplenial cortex; vPCC, ventral posterior cingulate cortex.

Irrespective of GSR, we consistently found increased connectivity between the right aMCC (area 24) and the left postcentral gyrus, the right dPCC and the right lingual, the left vPCC and the right middle occipital gyrus, the right vPCC and the bilateral precuneus, the left RSC and the right lingual gyrus, and the right RSC and the bilateral lingual gyrus and left parahippocampal gyrus (Figure 4).

### Volumetric atrophy of the cingulate subregions in schizophrenia

We also extracted GMV of each cingulate subregion and compared between the two groups. Compared with healthy subjects, schizophrenia patients had significantly reduced GMV (*P*<0.05, Bonferroni corrected) in all cingulate subregions except for the left (*P*=0.004, uncorrected) and right (*P*=0.014, uncorrected) RSCs (Table 2).

### The rsFC differences of the cingulate subregions after GMV correction

As done in a previous study,^32^ we also tested the effect of local GMV atrophy on the rsFC changes of the cingulate subregions. We compared intergroup differences in these rsFCs without and with GMV correction. Compared with rsFC results without GMV correction, most of the rsFCs with significant group differences remained significant after GMV correction, suggesting that rsFC alterations of the cingulate subregions were relatively independent on GMV atrophy.

### Correlations between rsFCs of the cingulate subregions and clinical parameters

We performed partial correlation analyses between the altered rsFCs and illness duration, Positive and Negative Syndrome Scale scores, and current antipsychotic dosage in chlorpromazine equivalents controlling for gender and age. We did not find any significant correlations between rsFCs of the cingulate subregions with the current antipsychotic dosage in chlorpromazine equivalents, the illness duration or the Positive and Negative Syndrome Scale scores.

## Discussion

To the best of our knowledge, this is the first study to systematically investigate rsFC changes of the cingulate subregions in schizophrenia. GSR greatly affected results of between-group comparisons in rsFCs of the cingulate subregions. Schizophrenia patients exhibited decreased rsFCs in most cingulate subregions after GSR, but only showed increased rsFCs without GSR. In schizophrenia, only some increased rsFCs were consistently present irrespective of GSR, which may challenge the functional disconnection hypothesis of schizophrenia. Because each cingulate subregion connects with its specific functional networks, we shall discuss our findings from the perspective of functional networks.

### Imaging sequence for resting-state fMRI

Most previous resting-state fMRI studies have used the EPI sequence to acquire functional imaging data; in this study, we adopted a SENSE-SPIRAL sequence, which has been proposed to be superior to the EPI sequence in terms of imaging quality.^24^ The SENSE is a parallel imaging method and has been used to shorten scan time and to reduce susceptibility-induced artifacts.^33^ We found that fMRI data acquired by SENSE-SPIRAL sequence exhibited less signal loss and distortion in the sACC and OFC compared with those acquired by EPI sequence. Consequently, fMRI data acquired by SENSE-SPIRAL sequence were used for rsFC analyses in the present study.

### GSR effects on between-group differences in rsFCs of the cingulate subregions

The global signal has been thought to reflect non-neuronal noise (for example, physiological, movement, scanner-related), which may induce artifactual high correlations across the brain. GSR has been used as a standard step during the processing of resting-state fMRI data^34^ and is thought to improve the anatomical specificity of rsFC findings via reducing the obscure effect of BOLD fluctuations of non-neuronal origin.^35, 36^ This is supported by findings that GSR can induce enhanced detection of system-specific correlations and improved correspondence between resting-state correlations and anatomy.^25^ Besides noise, global signal may reflect neurobiologically important information^37^ that is possibly altered in brain disorders. This is confirmed by a recent study reporting significantly increased global signal variability in schizophrenia but not in bipolar disorder.^27^ This suggests that global signal has neurobiological meaning and diagnostic specificity. Thus, in this study, both fMRI data with and without GSR were used in the rsFC analysis.

Consistent with previous studies with GSR that found functional disconnection in schizophrenia,^38, 39^ we found extensive functional disconnection in the cingulate subregions in schizophrenia with GSR. In contrast, without GSR, between-group difference patterns were qualitatively altered: we only found evidence for increased rsFC in schizophrenia, and no evidence for reductions. The similar GSR effect has also been reported in the prefrontal connectivity and thalamo-cortical connectivity in schizophrenia.^27^ The GSR effect on between-group difference patterns has been explained by a larger global signal variance in schizophrenia. GSR generally induces a relatively uniform transformation of the between-group effect without changing the topography of voxel-wise between-group differences.^27^ In this situation, statistical thresholding may lead to qualitatively distinct between-group inferences after GSR.

#### Altered connectivity of the sACC in schizophrenia

The emotional disturbance is a core feature of schizophrenia,^40, 41, 42^ and these patients also have deficits in emotion recognition.^43^ In our study, the sACC was specifically connected to the AN, which is known to be involved in mood regulation and affective processing.^44^ The sACC showed reduced connectivity with the OFC and amygdala after GSR, which suggests that the functional disconnection of the AN may contribute to emotional disturbance in schizophrenia. However, this reduced connectivity in schizophrenia was not present without GSR. Thus, we cannot exclude that the connectivity change may be a result of GSR.

#### Altered connectivity of the pACC in schizophrenia

The rsFC pattern of the pACC indicates that it is a hub region of the cingulate cortex and functionally connects to the AN, SN and DMN. We found that the pACC had reduced connectivity with the aMCC, fronto-insular cortex and striatum after GSR, which are core nodes of the SN.^45, 46^ The SN serves to identify salient stimuli and initiate appropriate cognitive manipulation to guide behavior. The pACC is a center for emotional processing; its functional disconnection with the SN may support a functional deficit in processing emotionally salient stimuli in schizophrenia.^47^ However, we also did not reproduce the finding in fMRI data without GSR, which is possibly caused by the effect of GSR.

#### Altered connectivity of the aMCC in schizophrenia

As an important node of the SN, the aMCC has been reported to be involved in a variety of cognitive control functions. Thus our finding of the reduced connectivity between the aMCC and the anterior insular cortex and striatum in schizophrenia after GSR may indicate functional disconnection within the SN, which has been reported in schizophrenia.^48^ Moreover, the impairment of the SN has been linked to clinical symptoms in schizophrenia, such as delusional thought, disorganization symptoms and psychomotor poverty syndrome.^46^ As a center of cognitive control, the aMCC has strong rsFC with the central executive network, which is mainly composed of the dorsolateral prefrontal cortex and posterior parietal cortex. We found that the area 32 of the aMCC has reduced connectivity with these two regions after GSR, suggesting that the functional disconnection between the cognitive control and execution networks may be related to cognitive deficits in schizophrenia. Because the reduced connectivity in schizophrenia was not present without GSR, we cannot determine whether it is a characteristic change in schizophrenia.

#### Altered connectivity of motor components of the MCC in schizophrenia

Both the aMCC and pMCC contain cingulate motor regions, which involve in sensorimotor integration and motor control. The impairments of the sensorimotor network have been reported in schizophrenia.^49, 50, 51^ In schizophrenia, we found reduced connectivity of the MCC with the SMA, posterior insular cortex, striatum and thalamus after GSR, which are components of the sensorimotor network and are deeply involved in sensorimotor integration. However, irrespective of GSR, the aMCC consistently exhibited increased connectivity with the left postcentral gyrus in these patients. These findings suggest that the rsFC changes of the cingulate motor areas are rather complex.

#### Altered DMN connectivity in schizophrenia

The dPCC, vPCC and RSC showed similar network connectivity patterns; they were densely connected with the DMN. The DMN impairment has been documented in schizophrenia.^52, 53^ This is consistent with our findings of the reduced connectivity of the left dPCC and the bilateral RSCs with the left precuneus and MPFC in schizophrenia patients after GSR. The DMN is associated with memory and self-referential processing; the impairment of which has been related to these functional deficits in schizophrenia patients.^54, 55^ With and without GSR, we consistently found increased connectivity of the PCC and RSC in schizophrenia. These findings suggest that DMN connectivity changes are important characteristics of schizophrenia.

There are several limitations of our study. First, schizophrenia patients were receiving different antipsychotic medication therapy. Although we did not reveal any significant correlations between the altered rsFCs and antipsychotic dosage, we cannot absolutely exclude the possibility of antipsychotic treatment effects on connectivity in a dose-independent way. Second, most patients in this study have chronic schizophrenia with mixed symptoms, which may influence our interpretation. Finally, we extracted the cingulate subregions on the basis of a previous study by manually defining the margin of each subregion. An *in vivo* parcellation of the cingulate cortex based on the rsFC patterns may result in a more accurate definition of each cingulate subregion.

In conclusion, we used an improved fMRI technique to detect rsFC alterations of the cingulate subregions in schizophrenia. We found that GSR greatly affected between-group inferences in rsFCs of the cingulate subregions. Schizophrenia patients exhibited decreased rsFCs in most cingulate subregions after GSR, but only showed increased rsFCs without GSR. The consistently increased rsFCs in cingulate subregions may challenge the functional disconnection hypothesis of schizophrenia.

## Figures and Tables

**Table 1:** Demographic and clinical characteristics of subjects

*Characteristic*	*Healthy controls (*N=*102)*		*Schizophrenia patients (*N=*94)*		*Analysis*		
	*Mean*	*s.d.*	*Mean*	*s.d.*	t*/*χ^*2*^	*df*	P
Age (years)	33.4	10.6	33.6	7.7	*t*=0.15	194	0.88
Gender (males:females)	45:57		52:42		*χ*^2^=2.46	1	0.12
Illness duration (months)			120.1	89.4			
							
*Positive and Negative Syndrome Scale*							
Positive subscore			16.6	7.9			
Negative subscore			20.3	8.8			
Current antipsychotic dosage (chlorpromazine equivalents, mg per day)			450.4	341.0			

**Table 2:** GMV changes between schizophrenia patients and healthy controls

*Cingulate* *subregions*	*Healthy controls* *(*N=*102)*		*Schizophrenia* *patients (*N=*94)*		*Analysis*	
	*Mean*	*s.d.*	*Mean*	*s.d.*	t	P
Left sACC	0.66	0.067	0.61	0.077	5.01	<0.001
Left pACC (area 24)	0.58	0.071	0.54	0.071	4.35	<0.001
Left pACC (area 32)	0.64	0.062	0.57	0.073	6.64	<0.001
Left aMCC (area 24)	0.54	0.066	0.50	0.059	3.83	<0.001
Left aMCC (area 32)	0.64	0.056	0.58	0.067	6.22	<0.001
Left pMCC	0.62	0.066	0.59	0.068	3.24	<0.001
Left dPCC	0.76	0.079	0.71	0.080	4.35	<0.001
Left vPCC	0.61	0.056	0.58	0.059	3.83	<0.001
Left RSC	0.44	0.46	0.42	0.053	2.95	0.004
Right sACC	0.62	0.055	0.58	0.067	5.28	<0.001
Right pACC (area 24)	0.56	0.084	0.52	0.076	3.18	0.002
Right pACC (area 32)	0.60	0.052	0.54	0.068	6.32	<0.001
Right aMCC (area 24)	0.54	0.058	0.50	0.064	4.35	<0.001
Right aMCC (area 32)	0.60	0.057	0.54	0.062	6.11	<0.001
Right pMCC	0.56	0.055	0.53	0.056	4.13	<0.001
Right dPCC	0.72	0.071	0.68	0.075	3.71	<0.001
Right vPCC	0.67	0.063	0.63	0.072	3.32	0.001
Right RSC	0.34	0.036	0.33	0.041	2.48	0.014

Abbreviations: aMCC, anterior mid-cingulate cortex; dPCC, dorsal posterior cingulate cortex; GMV, gray matter volume; pACC, pregenual anterior cingulate cortex; pMCC, posterior mid-cingulate cortex; RSC, retrosplenial cortex; sACC, subgenual anterior cingulate cortex; vPCC ventral posterior cingulate cortex.
