# Supplementary material for: Altered functional connectivity of the cingulate subregions in schizophrenia
Source: Transl Psychiatry. 2015 Jun 2;5(6):e575–. doi: 10.1038/tp.2015.69 (PMC4490280; doi:10.1038/tp.2015.69)
Supplement: Supplementary Figures [file tp201569x1.doc]

**SUPPLEMENTARY MATERIALS**


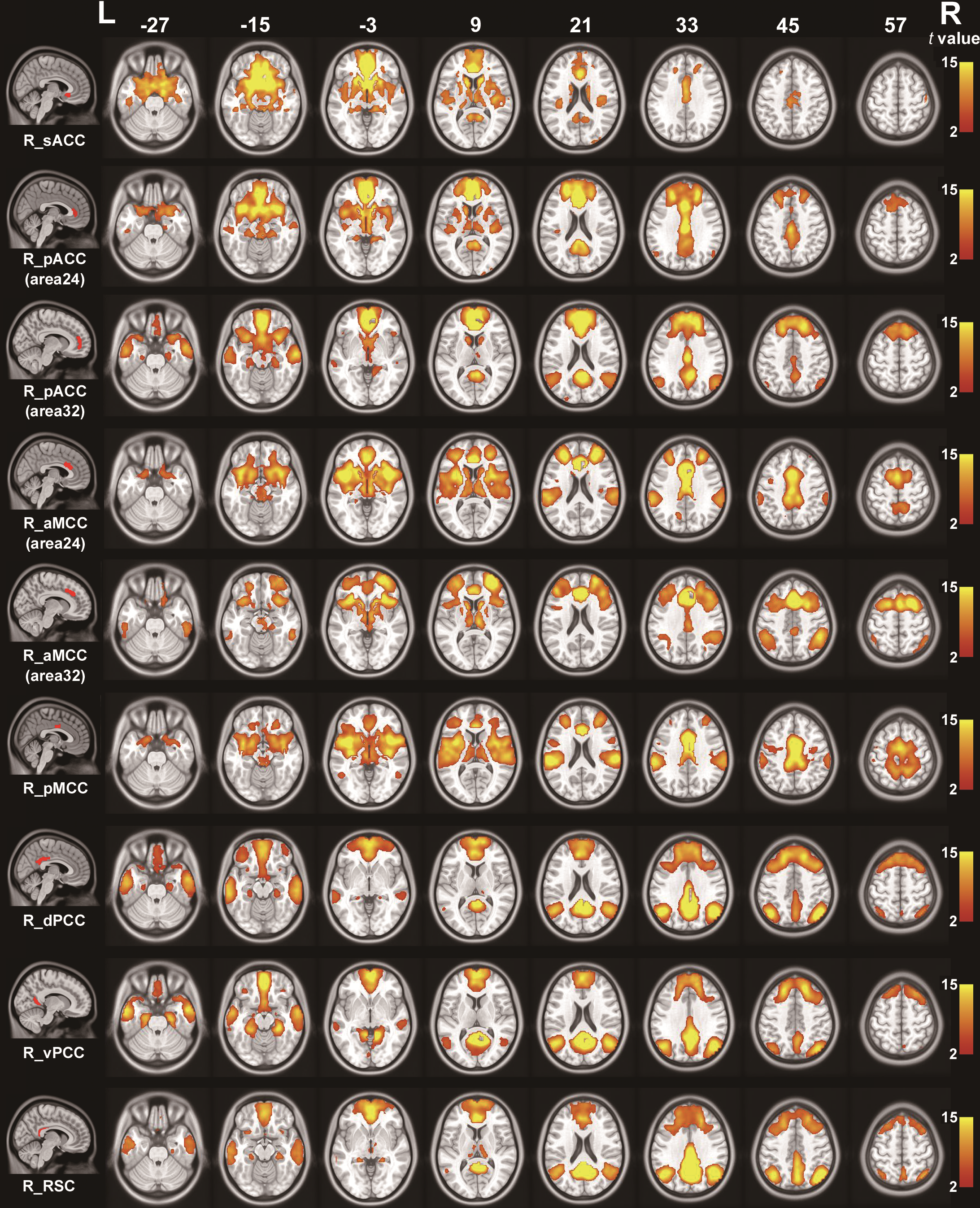


**Supplementary Figure 1. Resting-state functional connectivity maps of the right cingulate subregion in healthy controls with GSR.** Only positive connectivity map of each cingulate subregion is depicted. Multiple comparisons are corrected by a false discovery rate (FDR) with a significant threshold of *p* <0.05. L indicates left; R, right.

**
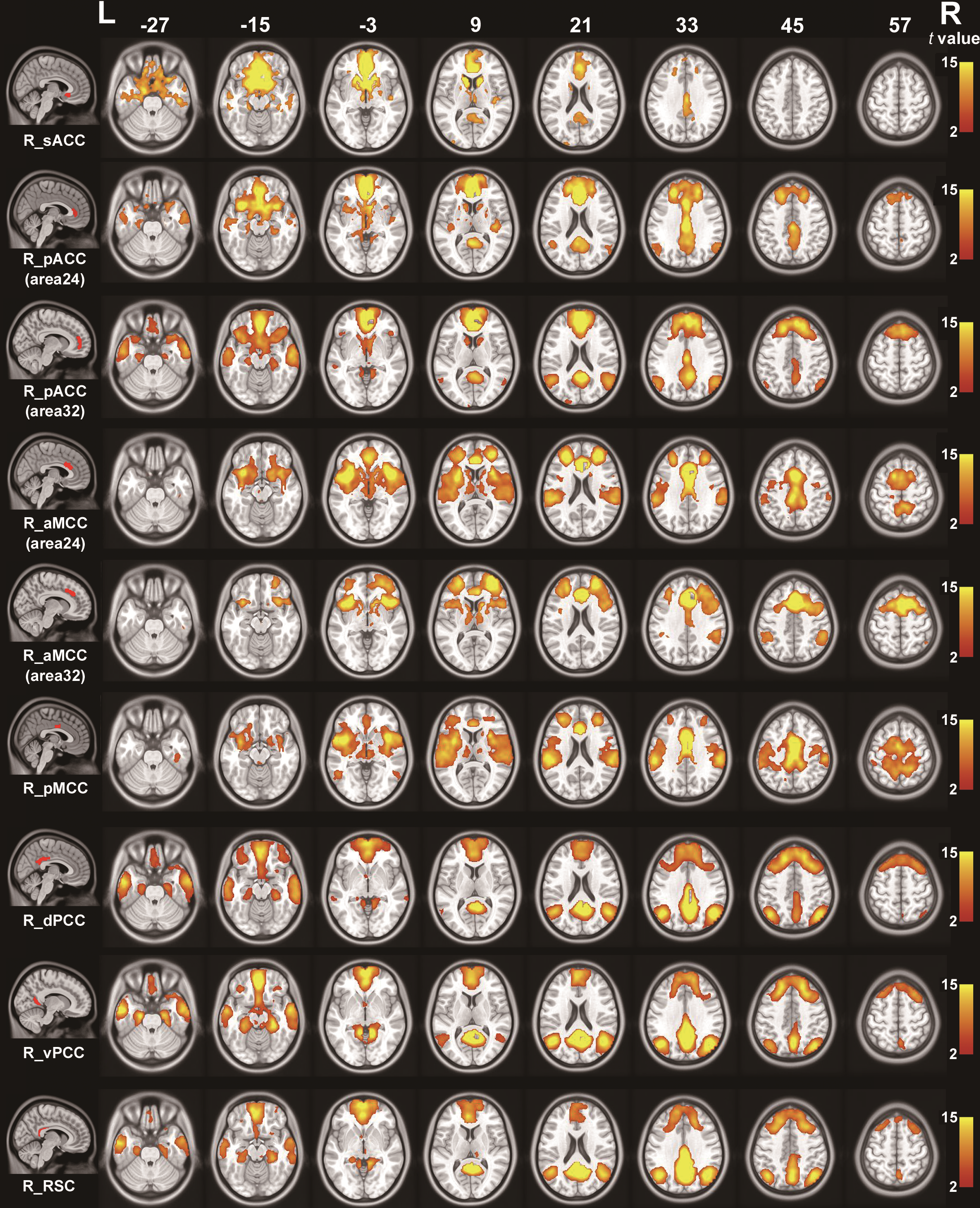
**

**Supplementary Figure 2. Resting-state functional connectivity maps of the right cingulate subregion in schizophrenia patients with GSR.** Only positive connectivity map of each cingulate subregion is depicted. Multiple comparisons are corrected by a false discovery rate (FDR) with a significant threshold of *p* <0.05. L indicates left; R, right.

**
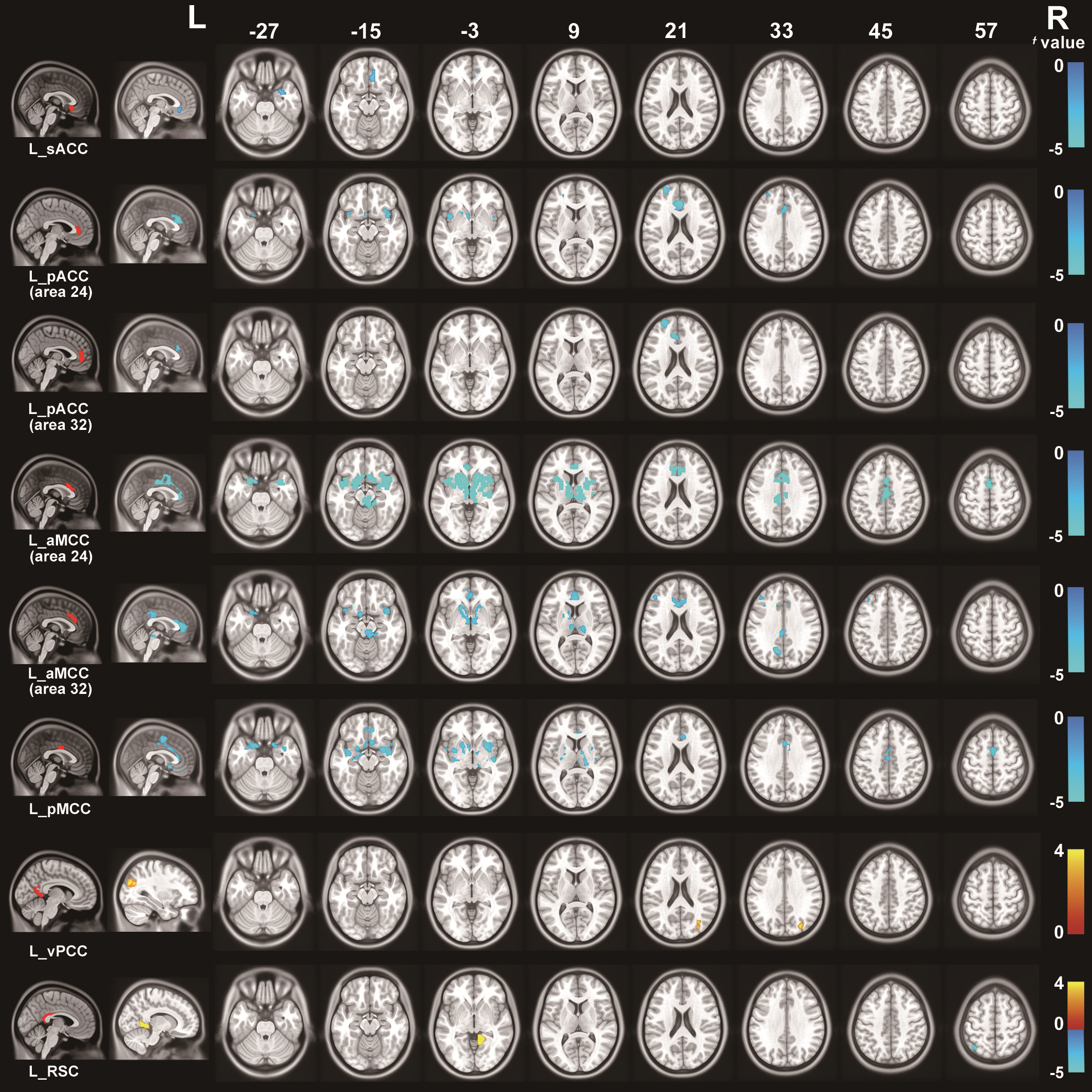
**

**Supplementary Figure 3. Altered resting-state functional connectivity of the left cingulate subregions in schizophrenia patients with GSR.** Warm color represents increased connectivity and cold color indicates reduced connectivity in schizophrenia patients. L indicates left, R, right.

**
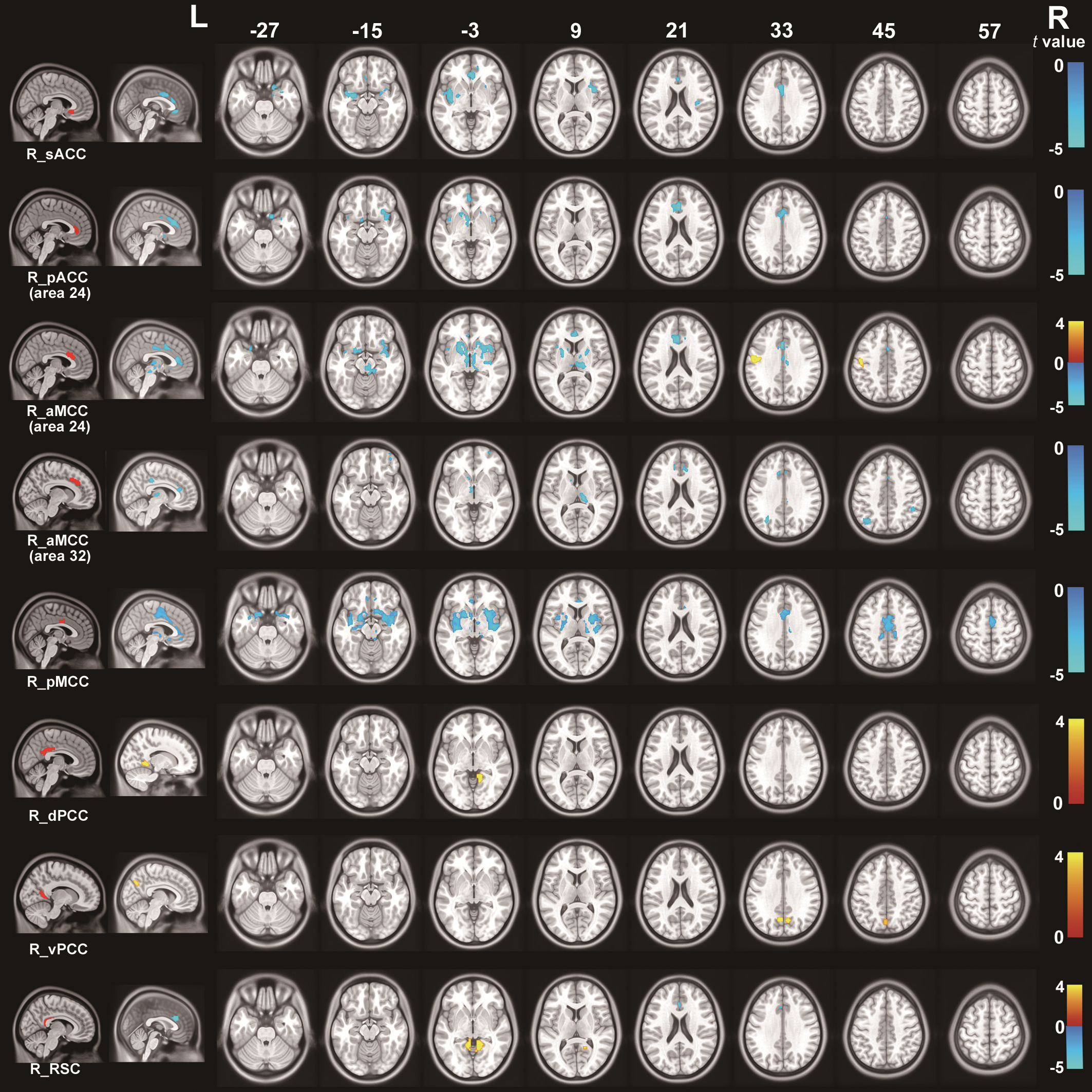
**

**Supplementary Figure 4. Altered resting-state functional connectivity of the right cingulate subregions in schizophrenia patients withGSR.** Warm color represents increased connectivity and cold color indicates reduced connectivity in schizophrenia patients. L indicates left, R, right.

**
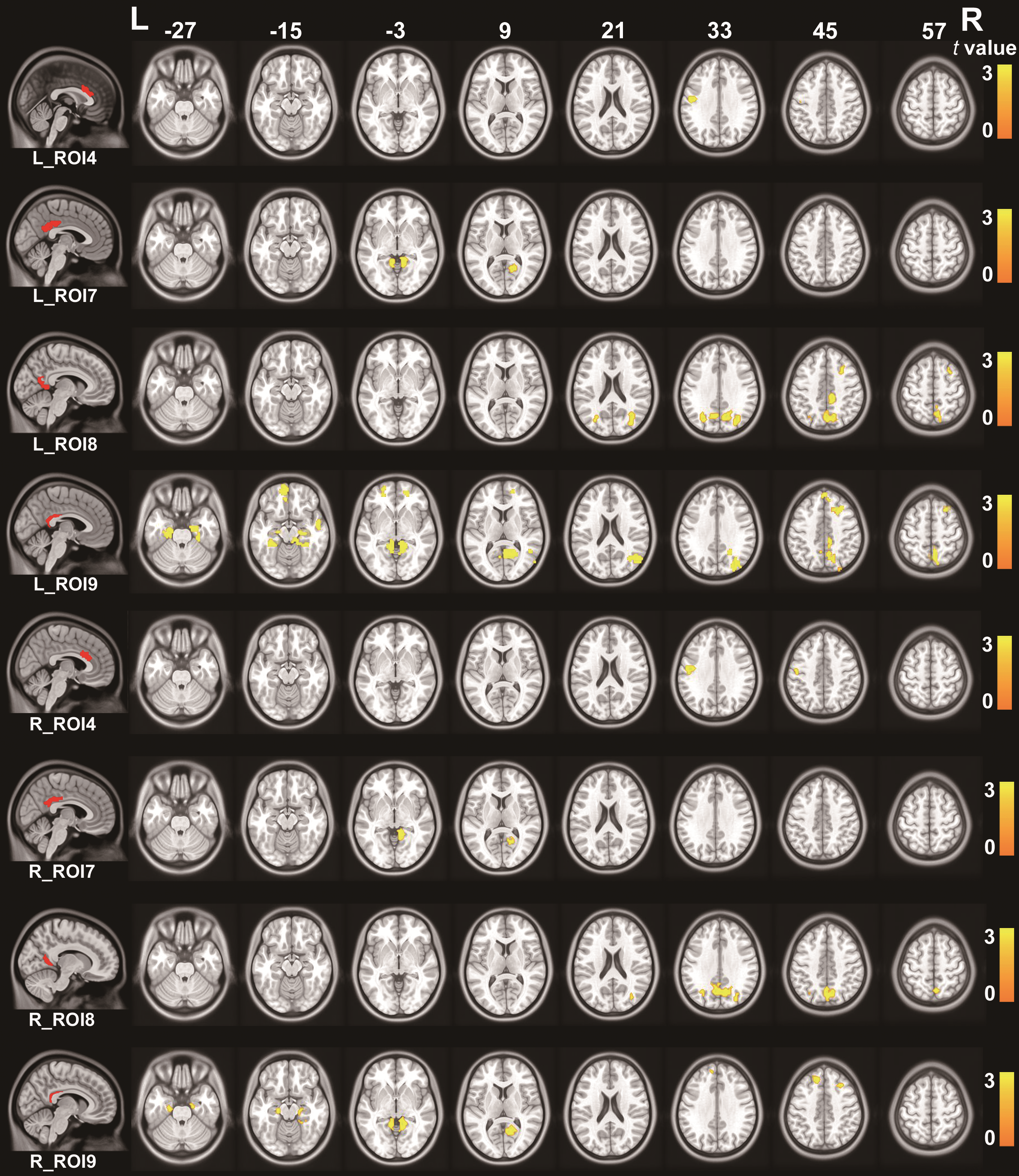
**

**Supplementary Figure 5. Altered resting-state functional connectivity of the bilateral cingulate subregions in schizophrenia patients withoutGSR.** Warm color represents increased connectivity. L indicates left, R, right.

**Supplementary Table 1. Functional connectivity changes in schizophrenia (with global signal regression)**

| Cingulate subregions | Connected brain regions^a^ | Montreal Neurological Coordinates (x,y,z) | Peak t-value | Cluster size(voxels)^b^ |
| --- | --- | --- | --- | --- |
| Left sACC | Right OFC | 6, 30, -15 | -4.87 | 51 |
|  | Right superior temopral pole | 36, 9, -21 | -4.54 | 32 |
| Left pACC (area 24) | Bilateral aMCC | 0, 33, 21 | -5.79 | 180 |
|  | Left insula | -39, 6, -6 | -4.16 | 89 |
|  | Right insula | 33, 9, -18 | -4.38 | 64 |
|  | Left caudate | -9, 9, 0 | -4.50 | 48 |
|  | Left middle frontal gyrus | -27, 57, 21 | -4.73 | 74 |
| Left pACC(area 32) | Left aMCC | -9, 33, 21 | -5.00 | 57 |
|  | Left prefrontal gyrus | -24, 60, 18 | -4.97 | 59 |
| Left aMCC(area 24) | Bilateral pACC and pMCC | -9, 18, 24 | -4.85 | 465 |
|  | Bilateral striatum, thalamus and insula | -9, 15, -12 | -5.51 | 1625 |
| Left aMCC(area 32) | Bilateral pACC and right aMCC | 6, 33, 15 | -5.00 | 291 |
|  | Left striatum and thalamus | -3, -21, 6 | -5.08 | 324 |
|  | Left insula | -39, 12, -15 | -3.70 | 33 |
|  | Right striatum, thalamus and insula | 12, 9, -6 | -5.55 | 289 |
|  | Left precuneus | -12, -60, 33 | -5.63 | 80 |
|  | Bilateral dPCC | -3, -30, 36 | -4.37 | 90 |
|  | Left middle frontal gyrus | -39, 27, 42 | -4.06 | 87 |
| Left pMCC | Bilateral SMA, pACC, and aMCC | -6, 0, 63 | -4.77 | 327 |
|  | Left putamen, insula, and superior temporal pole | -33, -18, -6 | -4.73 | 608 |
|  | Right striatum, insula, superior temporal pole and amygdala | 36, -9, -12 | -5.07 | 496 |
|  | Right thalamus | 18, -21,9 | -5.40 | 129 |
|  | Right OFC | 9, 36, -12 | -4.64 | 52 |
| Left vPCC | Right middle occipital gyrus | 30, -78, 30 | 4.46 | 40 |
| Left RSC | Right lingual gyrus | 12, -42, -6 | 5.37 | 79 |
|  | Left inferior parietal gyrus | -39, -66, 54 | -4.62 | 48 |
| Right sACC | Right MPFC | 3, 30,-6 | -5.32 | 116 |
|  | Left insula and amygdala | -30, -3, -15 | -4.88 | 192 |
|  | Right amygdala | 15, 9, -30 | -4.17 | 96 |
|  | Right insula | 36, 6, 9 | -4.40 | 49 |
|  | Bilateral aMCC and pMCC | -3,12, 33 | -4.25 | 111 |
| Right pACC (area 24) | Bilateral aMCC | 0, 33, 24 | -4.92 | 205 |
|  | Left insula | -39, 9, -6 | -4.08 | 46 |
|  | Right insula | 33, 9, -21 | -4.99 | 156 |
|  | Left OFC | -3, 42, 0 | -4.14 | 59 |
|  | Left striatum | -9, 9,0 | -4.92 | 109 |
| Right aMCC(area 24) | Bilateral pACC, pMCC, SMA, and left aMCC | 0, 18, 30 | -5.26 | 388 |
|  | Bilateral striatum, thalamus and right insula | -18, 0, 6 | -5.00 | 1135 |
|  | Left insula | -39, 6, -9 | -4.13 | 49 |
|  | Left postcentral gyrus | -51, -12, 42 | 5.21 | 81 |
| Right aMCC(area 32) | Right pMCC | 6, -33, 39 | -4.23 | 33 |
|  | Left middle frontal gyrus | -33, 24, 51 | -4.15 | 933 |
|  | Right middle frontal gyrus | 24, 18, 60 | -4.09 | 140 |
|  | Left angular gyrus | -33, -69, 30 | -4.97 | 97 |
|  | Right angular gyrus | 48, -45, 45 | -3.56 | 54 |
|  | Left thalamus | -6, -9, -3 | -4.06 | 40 |
|  | Right thalamus | 12, -24, 9 | -4.80 | 49 |
|  | Right OFC | 33, 54, -9 | -3.30 | 44 |
|  | Bilateral MPFC | 3, 18, 42 | -3.96 | 122 |
|  | Left caudate | -15, 18, 3 | -4.54 | 33 |
| Right pMCC | Bilateral SMA , pACC, aMCC, and left pMCC | 0, -3, 51 | -4.94 | 766 |
|  | Bilateral Striatum and insula | 36, -15, -3 | -6.07 | 1797 |
|  | Left thalamus | -9, -18, -6 | -3.15 | 33 |
|  | Right thalamus | 6, -39, -15 | -4.79 | 147 |
| Right dPCC | Right lingual gyrus | 12, -42, -3 | 5.15 | 31 |
| Right vPCC | Bilateral precuneus | -3, -69, 39 | 5.06 | 47 |
| Right RSC | Left MPFC | 0, 27, 27 | -4.56 | 56 |
|  | Left lingual gyrus | -9, -45, -6 | 5.57 | 38 |
|  | Right lingual gyrus | 12, -45, -3 | 5.60 | 96 |

^a^ The positive significant changes were observed in right aMCC(area 24) and vPCC, and the bilateral dPCC and RSC , and the other significant changes were observed in the schizophrenia patients < normal controls , with false discovery rate correction, *p*<0.05, cluster size>30 voxels.

^b^ Voxel size was 3×3×3mm^3^.

Abbreviations: aMCC, anterior mid-cingulate cortex; dPCC, dorsal posterior cingulate cortex; FIC, fronto-insular cortex; MPFC, medial prefrontal cortex; OFC, orbitofrontal cortex; pACC, pregenual anterior cingulate cortex; pMCC, posterior mid-cingulate cortex; RSC, retrosplenial cortex; sACC, subgenual anterior cingulate cortex; SMA, supplementary motor area; vPCC ventral posterior cingulate cortex.

**Supplementary Table 2. Functional connectivity changes in schizophrenia (without global signal regression)**

| Cingulate subregions | Connected brain regions^a^ | Montreal Neurological Coordinates (x,y,z) | Peak t-value | Cluster size(voxels)^b^ |
| --- | --- | --- | --- | --- |
| Left aMCC(area 24) | Left postcentral gyrus | -48, -9, 30 | 5.44 | 59 |
| Left dPCC | Right lingual gyrus | 12, -42, -3 | 5.96 | 129 |
|  | Left lingual gyrus | -9, -45, -6 | 6.57 | 31 |
| Left vPCC | Right precuneus and middle occipital gyrus | 30, -78, 30 | 4.84 | 626 |
|  | Right MCC | 12, -30, 42 | 5.73 | 61 |
|  | Right middle frontal gyrus | 30, 18, 48 | 3.80 | 53 |
| Left RSC | Right parahippocampal gyrus | 27, -30, -27 | 4.17 | 129 |
|  | Left parahippocampal gyrus | -33, -39, -15 | 5.55 | 141 |
|  | Right middle temporal gyrus | 51, -3, -18 | 3.69 | 34 |
|  | Left OFC | -12, 54, -21 | 3.89 | 138 |
|  | Right superior frontal gyrus | 18, 54, -9 | 4.49 | 48 |
|  | Right calcarine gyrus and left lingual gyrus | -12, -48, -3 | 6.48 | 271 |
|  | Right middle temporal gyrus | 30, -81, 30 | 4.79 | 214 |
|  | Right precuneus | 9, -36, 51 | 5.07 | 168 |
|  | Right middle frontal gyrus | 30, 24, 51 | 4.44 | 84 |
|  | Right superior frontal ghrus | 0, 51, 48 | 3.69 | 34 |
| Right aMCC(area 24) | Left postcentral gyrus | -48, -9, 33 | 5.44 | 99 |
| Right dPCC | Right lingual gyrus | 12, -42, -3 | 5.77 | 118 |
| Right vPCC | Right middle occipital gyrus | 27, -75, 36 | 3.65 | 48 |
|  | Bilateral precuneus | -3, -69, 39 | 5.33 | 338 |
|  | Left middle occipital gyrus | -33, -66, 36 | 4.22 | 32 |
| Right RSC | Right parahippocampal gyrus | 18, -27, -18 | 3.90 | 44 |
|  | Left parahippocampal gyrus | -21, -15, -21 | 5.08 | 72 |
|  | Right lingual gyrus | 12, -45, -3 | 6.04 | 255 |
|  | Left superior frontal gyrus | -12, 33, 45 | 4.51 | 50 |
|  | Right middle frontal gyrus | 30, 24, 48 | 3.64 | 33 |

^a^ The positive significant changes were only observed, in bilateral aMCC(area 24), vPCC, dPCC and the RSC in the schizophrenia patients > normal controls , with false discovery rate correction, *p*<0.05, cluster size>30 voxels.

^b^ Voxel size was 3×3×3mm^3^.

Abbreviations: aMCC, anterior mid-cingulate cortex; dPCC, dorsal posterior cingulate cortex; OFC, orbital frontal cortex; RSC, retrosplenial cortex; vPCC ventral posterior cingulate cortex.
